# The association between the diversity of online activities on smartphones and cognitive function among middle-aged and elderly Chinese adults

**DOI:** 10.1186/s12889-024-17932-0

**Published:** 2024-02-21

**Authors:** Qian Chen, Haoqiang Ji, Qingxin Shang

**Affiliations:** 1 College of Chinese Medicine, Shandong University of Traditional Chinese Medicine, 250300 Jinan, Shandong Province China; 2 School of Public Health, Shandong University, 250012 Jinan, Shandong Province China; 3 Shandong University, 44 West Wenhua Road, Lixia District, Jinan City, Shandong Province China; 4 Shandong University of Traditional Chinese Medicine, 4655 Daxue Road, Changqing District, Jinan City, Shandong Province China

**Keywords:** Smartphone, Online activity, Cognitive function, The middle-aged and elderly, China

## Abstract

**Background:**

Many studies have shown that using smartphones can improve cognitive function, but no studies have shown the effect of the diversity of online activities on cognitive function. Therefore, this study explores the association between the diversity of online activity on smartphones and cognitive function among middle-aged and elderly Chinese adults.

**Methods:**

A total of 13,347 Chinese middle-aged and elderly participants were used in the final analysis. Multivariate linear regression models were used to explore the relationships among the frequency of smartphone use, number of online activities, various activities, and cognitive function.

**Results:**

We found that 2,143 respondents (16.1%) used smartphones, and the top three online activities were watching news (80.3%), posting moments (72.4%), and chatting (68.0%) among all smartphone users to access the internet. After adjusting for all covariates, we found that the increase in the frequency of smartphone use and the number of online activities were correlated with a higher cognitive score. Moreover, some online activities, such as watching news (β:0.5, 95% CI:0.2–0.8), posting moments (β:0.4, 95% CI:0.2–0.7) playing games (β:0.3, 95% CI:0.03–0.6) and making mobile payments (β:0.3, 95% CI:0.1–0.5) were independently associated with good cognitive function.

**Discussion:**

In the middle-aged and elderly population, smartphone use plays an important role in cognitive function. Considering the increasing prevalence of smartphones among middle-aged and elderly individuals, this study can provide references and insights for health education and in-depth scientific research related to internet usage.

## Background

As one of the most populous countries, China also has a large middle-aged and elderly population [1]. In 2020, China had a total population of 1.41 billion, with middle-aged and elderly people accounting for 42.62% of the total [2]. As the ageing process accelerates, this upward trend indicates that China is likely to face an increased burden of age-related diseases, such as cognitive impairment. Cognitive decline may be an important risk factor for death and disability and a precursor to dementia [3]. Meanwhile, older adults with severe cognitive impairments experience a wide range of difficulties in their daily lives, such as memory loss, confusion about time or place, and challenges with problem solving. They require intensive care and their caregivers are also reported to experience more stress than other caregivers [4]. The gradual deterioration of cognitive function causes serious psychological distress and imposes an additional economic burden on both families and society [5]. Studies have found that change in cognitive function may be associated with social development and internet usage [6–9]. Therefore, investigating the relationship between the two could provide valuable insights for future intervention research and cognitive management among middle-aged and elderly people.

With the development of technology, the internet and smartphones are gradually playing a more important role in social activities. During the COVID-19 pandemic, the number of internet users grew rapidly in China, with 986 million internet users at the end of 2020. Although almost all of these internet users are also smartphone users (99.7%), smartphone ownership rates are not high among middle-aged and elderly people [10]. There is growing evidence that smartphones may provide a platform for cognitively stimulating activities, and thus might help slow cognitive decline during the process of normal ageing [6–8]. These studies seem to support the cognitive exercise hypothesis, suggesting that engaging in more stimulating activities can lead to better performance in cognitive tasks, possibly due to the ability of these stimuli to increase neurotransmitter levels and gray matter volume [11, 12]. A randomized controlled trial showed that older adults can learn smartphone-based memory strategies to improve their prospective memory function (improved by 2.5 times after the intervention) [9]. However, some studies have not found a positive relationship between smartphone use and cognitive function. A prospective cohort study found that smartphone use was associated with a reduction in response time (β =-0.03) but did not affect cognitive function in Australia [13]. A study found that longer smartphone use is associated with lower cognitive scores (24.6 points), while scores below 1 h of usage per day were recorded at 26.8 points [14]. The inconsistent results of previous research may be due to different research populations and significant spatial heterogeneity across different countries in terms of economy, society, and education. It could also be attributed to variations in the cognitive definitions and research designs employed in different studies. In addition, there is a lack of research on the impact of diverse online activities on cognition, and further studies in various regions are needed. This is because studying the effects of diverse online activities on cognition can help accurately identify beneficial activities and facilitate the development of targeted interventions such as health education and optimization of smartphone features.

With the popularity of smartphones among middle-aged and older adults, online activities may be affecting the cognition of this population, suggesting that more research should be done on this population to intervene in cognitive decline, not just limited to adolescents [15, 16]. Currently, there is insufficient research on the relationship between smartphone usage and cognitive function in middle-aged and elderly individuals, particularly within the Chinese population. To supplement the research evidence in China, we used data from the 2018 wave of the China Health and Retirement Longitudinal Study (CHARLS) to examine the association between the details of smartphone use and cognitive function among middle-aged and elderly Chinese adults (45 + years). Based on the literature review, this study proposes the following research hypotheses: (1) There is a positive correlation between higher smartphone usage frequency and higher cognitive scores. (2) There is a positive correlation between engaging in a greater variety of online activities and higher cognitive performance. (3) Engaging in internet activities such as chatting, watching news, watching videos, playing games, financial management, mobile payments, and posting WeChat moments on smartphones are associated with better cognitive performance.

## Methods

### Study sample

The CHARLS is a nationally representative longitudinal survey of Chinese people aged 45 years and older, including socioeconomic status, healthy lifestyle, health conditions, and many other variables, to serve the needs for scientific research on elderly individuals. The national baseline survey was conducted in 2011, with wave 2 in 2013, wave 3 in 2015, and wave 4 in 2018 [17]. To ensure sample representativeness, a multistage, stratified sampling strategy was used to select 19,816 participants from 150 counties/ districts of 28 provinces in 2018. The project obtained data through face-to-face household surveys assisted by community workers. This study used cross-sectional data from 2018, and the data can be accessed through the official website (http://charls.pku.edu.cn/). Among the 19,816 surveyed participants, this study extracted 13,347 participants according to the following criteria: (1) aged 45 and older and (2) no missing items in the study variables.

### Smartphone use

Smartphone use was defined as participants using cell phones to surf the internet in the last month. Frequency is how often smartphone users used their phones to surf the internet in the last month. In addition, the CHARLS collected 7 types of online activities from users using their smartphones, including chatting, watching the news, watching videos, playing games, financial management, mobile payments, and posting WeChat moments.

### Cognitive function

The study examined two composite measurements of cognitive functions: mental status and episodic memory. Mental status includes the participants’ orientation and numerical, visual, and spatial abilities, while episodic memory includes adults’ memory for autobiographical events, which is captured by their immediate memory and delayed memory [17].

Mental status was assessed by time orientation, numerical ability, and figure drawing test. The test of time orientation required the participants to recall the current date (year, month, day), the day of the week, and the current season. The time orientation score was equal to the number of correct answers to the 5 questions, ranging from 0 to 5. The numerical ability required the respondents to perform 7 serial subtractions starting from 100 (up to 5 times), and the total score was equal to the number of correct calculations, ranging from 0 to 5. However, this total score was reduced by half if the participants used paper, pen, or another aid to complete this assessment. The figure drawing test asked the participants to successfully reproduce a picture of two overlapping pentagons shown by the interviewers. The respondents who successfully drew this picture received 1 point, but those who failed to draw it received no score [18]. Finally, the mental status score was defined as the sum of all 3 parts, ranging from 0 to 11 [19].

To measure episodic memory, the interviewers read 10 Chinese words and asked the participants to repeat the words that they remembered (immediate recall). In addition, they were asked to recall the 10 words 5 min later (delayed recall) and were given 1 point for each word they recalled correctly. The score for episodic memory was equal to the average of the sum of immediate recall and delayed recall, ranging from 0 to 10 [18].

Finally, global cognition is considered to be a comprehensive cognitive ability that includes all aspects of mental status and episodic memory. Global cognition was defined as the sum of the scores for mental status and episodic memory, ranging from 0 to 21 [19].

### Covariates

In linear regression models, we adjusted for three types of covariates: demographic characteristics, health behaviour and health status. First, demographic characteristics included age (the meddle-aged: 45–59/ the elderly individuals: ≧60), gender (male/ female), residential area (urban area/ rural area) educational level (lower educational level: no senior middle school degree/ higher educational level: senior middle school degree or above), and marital status (married/ others: divorced, widowed, separated and never married). In addition, health behaviour included smoking status (current smoker/ former smoker/ never smoker), drinking status (never/ occasionally/ everyday) and sleep time (< 6 h/ 6-8 h/ >8 h). Finally, health status included the number of chronic diseases (no/ 1 type/ 2 types/ 3 types or more) and self-rated health (good: good and very good/ fair/ poor: poor and very poor).

### Statistical analysis

This study utilized an ecological research method to examine the association between the diversity of online activities on smartphones and cognitive function. The sample characteristics are described as the mean standard deviation (SD) for the continuous variables or number (%) for categorical variables according to smartphone use. T tests (for continuous variables) and Pearson’s chi-square tests (for categorical variables) were applied to compare the differences in basic characteristics between smartphone users and smartphone nonusers.

In multivariate linear regression models, the variance inflation factors (VIFs) of the three variables (smartphone use, frequency of use, and variety) are less than 10. Therefore, we adjusted all covariates (age, gender, residential area, educational level, marital status, smoking status, drinking status, sleep time, self-rated health status, and the number of chronic diseases) for these three variables to verify the relationship between them and cognitive function (global cognition, mental status, and episodic memory).

In addition, to deeply explore the association between cognitive function and variety of smartphone use, we added 7 types of online activity as key independent variables to the regression equation after adjusting for all covariates. Two-sided *P* < 0.05 was considered statistically significant. All data processing and analyses were performed in STATA version 14.0 (StataCorp LLC, College Station, Texas, USA) and R 4.0.3 (R Foundation for Statistical Computing).

## Results

### Characteristics

A total of 13,347 participants were included in the analysis, but only 2143 participants (16.1%) used smartphones to surf the internet. Almost half of the participants (50.6%) were female; 7358 (55.1%) of the participants were elderly; most (71.6%) of the elderly lived in rural areas; and a small number (15%) of the participants had a high school education or higher. Compared to the participants who did not use a smartphone, the smartphone users were more likely to be male, younger, live in urban areas, have a higher education level, be married, never drink, have proper sleep time, have no disease, and have good health status (*P* < 0.01 for all). Table 1 shows more details of the characteristics according to smartphone use.

**Table 1:** Characteristics of participants according to smartphones use

Characteristics	Total Sample (*N* = 13,347, %)	Smartphone use		P value
		No (*N* = 11,204, %)	Yes (*N* = 2143, %)	
**Global cognition** (mean ± SD)	12.6 ± 3.3	12.2 ± 3.2	14.9 ± 2.4	< 0.001
**Mental status** (mean ± SD)	8.3 ± 1.9	8.1 ± 1.9	9.4 ± 1.5	< 0.001
**Episodic memory** (mean ± SD)	4.3 ± 1.9	4.1 ± 1.9	5.5 ± 1.5	< 0.001
**Female**	6759 (50.6)	5784 (51.6)	975 (45.5)	< 0.001
**The elderly individuals**	7358 (55.1)	6715 (59.9)	643 (30)	< 0.001
**Rural area**	9554 (71.6)	8587 (76.6)	967 (45.1)	< 0.001
**High education level**	2006 (15)	1158 (10.3)	848 (39.6)	< 0.001
**Married**	10,878 (81.5)	9079 (81)	1799 (83.9)	0.001
**Smoking status**				0.485
Never	7670 (57.5)	6449 (57.6)	1221 (57)	
Former	1979 (14.8)	1672 (14.9)	307 (14.3)	
Current	3698 (27.7)	3083 (27.5)	615 (28.7)	
**Drinking status**				< 0.001
Never	8487 (63.6)	7434 (66.4)	1053 (49.1)	
occasionally	1102 (8.3)	778 (6.9)	324 (15.1)	
everyday	3758 (28.2)	2992 (26.7)	766 (35.7)	
**Sleep time (h)**				< 0.001
6–8	7835 (58.7)	6373 (56.9)	1462 (68.2)	
<6	4465 (33.5)	3874 (34.6)	591 (27.6)	
>8	1047 (7.8)	957 (8.5)	90 (4.2)	
**Number of chronic diseases**				< 0.001
0	2753 (20.6)	2177 (19.4)	576 (26.9)	
1	3176 (23.8)	2617 (23.4)	559 (26.1)	
2	2696 (20.2)	2305 (20.6)	391 (18.2)	
≧ 3	4722 (35.4)	4105 (36.6)	617 (28.8)	
**Self-rated Health**				< 0.001
Good	3406 (25.5)	2610 (23.3)	796 (37.1)	
Fair	6699 (50.2)	5609 (50.1)	1090 (50.9)	
Poor	3242 (24.3)	2985 (26.6)	257 (12)	

### Online activities of smartphone users

**Fig. 1:**
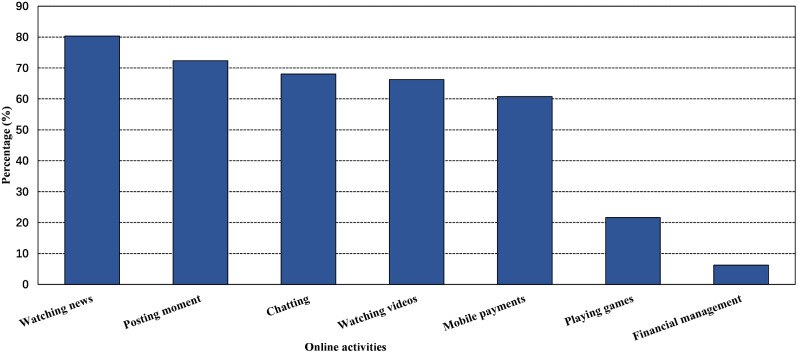
Online activities of smartphone users

Among all smartphone users used their phones to access the internet, the top three online activities were watching news (80.3%), posting moments (72.4%), and chatting (68.0%). Only 137 users used smartphones for financial management. Figure 1 shows more information about the online activities of smartphone users.

### Association between cognitive function and smartphone use

In the multivariate analysis of linear regression models, after controlling for potential confounders, smartphone use was a significant predictor of global cognition (β = 1.2, 95% CI = 1.1–1.4), mental status (β = 0.6, 95% CI = 0.5–0.7) and episodic memory (β = 0.6, 95% CI = 0.5–0.7). Meanwhile, a higher frequency of smartphone use is positively associated with better cognitive function. In addition, compared to adults who had no online activity, a greater variety of online activities was significantly associated with higher cognitive function (Table 2).

**Table 2:** The association between Smartphone usage and cognitive function

Smartphone usage		Global cognition			Mental status			Episodic memory	
		β (95%CI)	Р Value		β (95%CI)	Р Value		β (95%CI)	Р Value
Smartphone use									
No		Reference	Reference		Reference	Reference		Reference	Reference
Yes		1.2 (1.1–1.4)	< 0.001		0.6 (0.5–0.7)	< 0.001		0.6 (0.5–0.7)	< 0.001
**Frequency**									
No		Reference	Reference		Reference	Reference		Reference	Reference
Irregular		1.2 (0.9–1.5)	< 0.001		0.5 (0.3–0.7)	< 0.001		0.7 (0.5–0.9)	< 0.001
Daily		1.2 (1.1–1.4)	< 0.001		0.6 (0.5–0.7)	< 0.001		0.6 (0.5–0.7)	< 0.001
**Variety**								Reference	
0 type		Reference	Reference		Reference	Reference			Reference
1–3 type(s)		1.2 (1.0-1.4)	< 0.001		0.6 (0.5–0.7)	< 0.001		0.6 (0.5–0.7)	< 0.001
4 types or more		1.3 (1.1–1.5)	< 0.001		0.6 (0.5–0.7)	< 0.001		0.7 (0.5–0.8)	< 0.001

*Note* In the models predicting global cognition, all Adjusted R^2^ > 0.25 and all MSE < 2.833; in the models predicting mental status, all Adjusted R^2^ > 0.174 and all MSE < 1.754; In the models predicting Episodic memory, all Adjusted R^2^ > 0.206 and all MSE < 1.699

### The association between cognitive function and the number of online activities

**Fig. 2:**
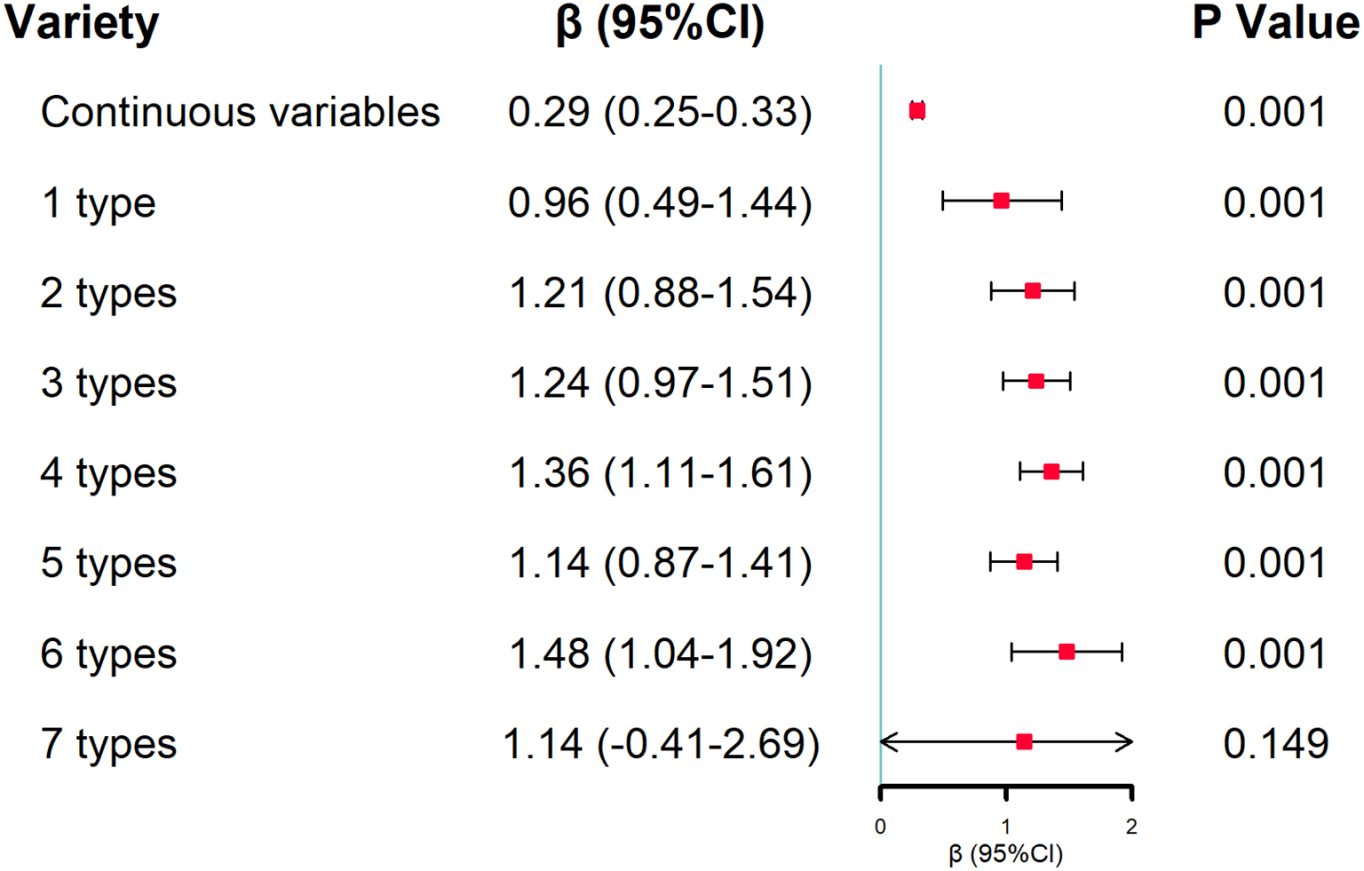
The association between cognitive function and the number of online activities. Note: To explore the effects of the number of online activities on cognitive function, we adjusted for gender, age, residential area, educational level, married status, smoke status, drinking status, sleep time, self-rated health, and the number of chronic diseases in the multivariable linear regression model. (Adjusted R^2^:0.248; MSE: 2.838)

After adjusting for all the covariates, compared with adults who had no online activity, the participants’ cognitive function improved as the number of online activities increased, but the 7 online activities were associated with good cognitive function at a 15% statistical level. This may be due to the small sample size of 7 online activities. The details of the association between cognitive function and a variety of smartphone online activities are shown in Fig. 2.

### Association between cognitive function and variety of online activities

**Fig. 3:**
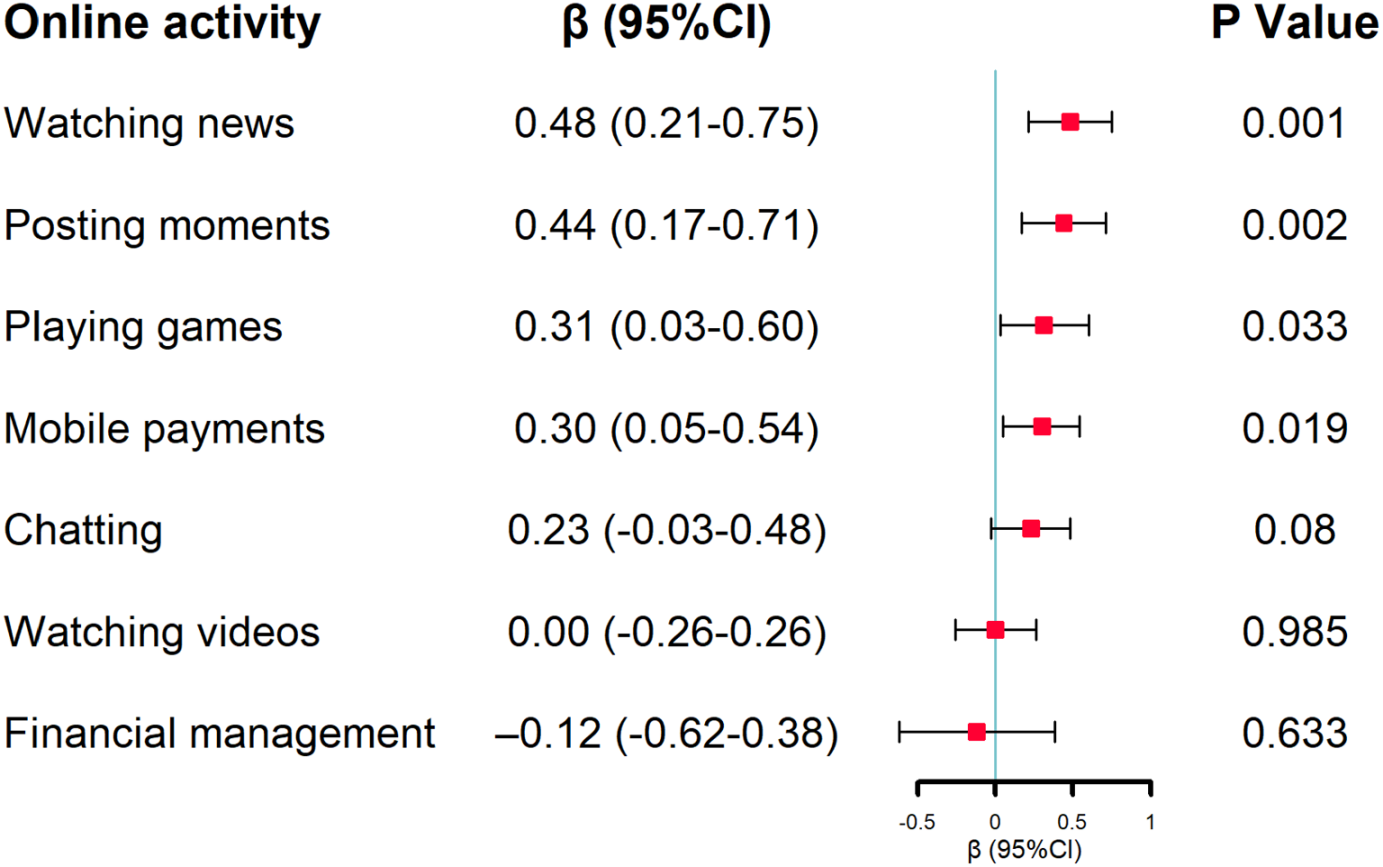
The association between different online activities and cognitive function. Note: To explore the effects of different online activities on cognitive function, we adjusted for gender, age, residential area, educational level, married status, smoke status, drinking status, sleep time, self-rated health, and the number of chronic diseases in the multivariable linear regression model. (Adjusted R^2^:0.238; MSE: 2.86To explore the effects of different online activities on cognitive function, we adjusted for gender, age, residential area, educational level, married status, smoke status, drinking status, sleep time, self-rated health, and the number of chronic diseases in the multivariable linear regression model. (Adjusted R^2^:0.238; MSE: 2.86

After adjusting for all the covariates, watching news (β:0.5, 95% CI:0.2–0.8), posting moments (β:0.4, 95% CI:0.2–0.7), playing games (β:0.3, 95% CI:0.03–0.6) and making mobile payments (β:0.3, 95% CI:0.1–0.5) were independently associated with good cognitive function, and chatting was associated with good cognitive function at the 10% statistical level. The other details are shown in Fig. 3.

## Discussion

This study, to our knowledge, is the first nationally representative study to explore the effects of the diversity of online activities and specific online activities on cognitive function among middle-aged and elderly Chinese adults. We found that few people (16.1%) have smartphones to access the internet and participating in online activity, and rural people have lower rates of use. They tend to go online for basic social and entertainment needs, such as watching news, posting moments, chatting, etc. In addition, after adjusting for all covariates, we found that individuals who use smartphones and those who use them daily scored higher in cognitive function. The increase in online activities on smartphones was correlated with higher cognitive scores (including mental status and episodic memory). Moreover, some online activities, such as chatting, playing games, posting moments, and watching news, were independently associated with good cognitive function.

Smartphone usage among Chinese residents still needs to increase, especially in rural areas. This phenomenon is closely related to China’s economic development. Rural areas are still behind in development. Villagers tend not to have high education levels, and their awareness of learning is insufficient, which indeed limits the promotion of smartphones in rural areas [20, 21]. In addition, women use smartphones less than men. There may be only one smartphone in each family, which is usually used by men due to the leadership of men in the family. Considering the functions and effects of smartphones, this gap should be changed.

This study found that the frequency of smartphone use is related to cognitive function. This is consistent with previous research suggesting that proper use of smartphones can potentially significantly improve cognitive function [22, 23]. Nevertheless, it is necessary to prevent internet addiction. Continuous and intensive daily use of mobile phones appears to have a negative impact on cognitive function. Frequent use of smartphones can lead to a lack of physical activity and a decline in physical fitness, including weakened grip strength and decreased flexibility (all *P* < 0.05) [24]. Individuals who are heavily addicted to the internet perform worse in terms of working memory capacity, visual reaction time, auditory reaction time and slow development of cognitive areas of the cortical surface (all *P* < 0.01) [25–27]. Therefore, health education regarding appropriate smartphone usage must be prioritized to mitigate the adverse health consequences of internet addiction. It is necessary to explore the dose‒response relationship between smartphone use time and cognitive function in future studies. Moreover, more online activity tends to lead to better cognitive function, which may be related to the combined effect of multiple network activities on the brain [6, 28].

This study found that people who watched news on their phones had higher cognitive function. Frings et al. utilized functional magnetic resonance imaging (fMRI) and found that watching news can strongly activate brain regions responsible for semantic and episodic memory processing, thereby promoting the development of neurons in the left lateral temporal cortex [29]. Individuals who regularly share their life updates on WeChat Moments exhibit higher cognitive function. This behavior may involve emotional expression, social interaction, and personal cognition, potentially activating brain regions related to emotion such as the limbic system and amygdala. It could also stimulate the brain’s reward system, leading to increased activity in dopaminergic neurons, which may improve emotional regulation and social cognitive abilities [30]. Additionally, it may activate neural networks in the prefrontal cortex related to self-understanding and personal cognition [31]. This study found that playing games is also associated with higher cognitive function. This may continuously activate and strengthen the relevant neural functions in the brain, such as the frontal lobe and parietal lobe, thereby enhancing spatial visualization and logical thinking abilities [32]. However, smartphone users should also be aware of the duration and type of games they play [33]. Violent and vulgar games may impair players’ cognitive control, and these games should be banned [34]. People who use mobile payments tend to have better cognitive function. Mobile payment may involve multiple cognitive functions, including attention, decision-making, and working memory. These behaviors may stimulate and enhance connectivity between neurons in regions such as the frontal lobe, parietal lobe, and temporal lobe, promoting neuroplasticity and improving brain structure and cognitive function [35]. Currently, most neurobiological mechanisms are based on hypotheses and speculations, and further research is needed to delve into the mechanisms of how network activity can modulate cognitive functions. In conclusion, there is a large amount of information contained on the internet, and smartphone users should pay attention to choosing beneficial online activities when using it.

Given the large number of middle-aged and elderly people in China and the important role of smartphones, smartphones will have great business potential in this group. The popularization of smartphones plays an important role in improving cognitive function and preventing dementia, so the use of smartphones in middle-aged and elderly individuals should be accelerated. This research can potentially facilitate the implementation and advancement of a nationwide smartphone popularization program, and government departments can collaborate with smartphone manufacturers to sell smartphones in bulk to this group through discount schemes. Meanwhile, during the COVID-19 pandemic, some functions of smartphones were particularly important and convenient, such as epidemiological investigation, nucleic acid test registration, access to various places, etc. Producing smartphones tailored for the elderly is a trend, and smartphones that are easy to operate, have large fonts, loud sound, and long battery life can help this group to adapt more quickly [36, 37].

### Limitations

The study had several limitations. First, given the cross-sectional design of the study, the results are not causal. Future causality should be explored in future longitudinal studies. Second, due to the different mobile internet environments, these associations may be different in other countries and areas. Therefore, the results apply to only middle-aged and elderly Chinese population. Third, there is no detailed record of the amount of time spent online each day, so we used the number of days spent online each week for calculation. The dose‒response relationship between daily smartphone use time and cognitive function should be fully explored in future studies. Fourth, the secondary data used may have poor timeliness, which is also one of the limitations of this study. Finally, cognitive function was assessed only by time orientation, numerical ability, figure drawing test and episodic memory, and other important aspects may not have been measured.

## Conclusions

In the study, only a few people had smartphones to access the internet and participate in online activity, and rural people had lower rates of use. The top three online activities were watching news, posting moments, and chatting among all those who used a smartphone to access the internet. Compared to individuals who do not use smartphones, those who use smartphones tend to have better cognitive conditions. Individuals who have higher frequency of smartphone usage and engage in more online activities tend to have better cognitive conditions. Moreover, some online activities, such as chatting, playing games, posting moments, and watching news, were independently associated with good cognitive function. Considering the usage rate and role of smartphones, the research and development of smartphones suitable for this population have good commercial prospects and public health significance. At the same time, middle-aged and elderly individuals should gradually adapt to online activities on smartphones and form civilized online habits, such as how to avoid web addiction and understanding legal boundaries, so that smartphones become a tool to facilitate life and promote cognitive function.

## Data Availability

The datasets used can be accessed through the official website (http://charls.pku.edu.cn/).
